# Zur Freiverantwortlichkeit der Entscheidung für einen assistierten Suizid

**DOI:** 10.1007/s00115-022-01386-z

**Published:** 2022-09-12

**Authors:** Henning Saß, Clemens Cording

**Affiliations:** 1 Klinik für Psychiatrie, Psychotherapie und Psychosomatik, Universitätsklinikum der RWTH Aachen, Pauwelsstr. 30, 52074 Aachen, Deutschland; 2 Klinik für Psychiatrie und Psychotherapie der Universität Regensburg am Bezirksklinikum, Regensburg, Deutschland

**Keywords:** Suizidassistenz, Begutachtung, Forensische Psychiatrie, Freie Willensbestimmung, Suizidprävention, Assistance for suicide, Assessment, Forensic psychiatry, Free will determination, Suicide prevention

## Abstract

Das Urteil des Bundesverfassungsgerichts zur Suizidassistenz hat zu lebhaften Debatten in der Medizin, im Deutschen Bundestag und in der Zivilgesellschaft geführt. Zentrale Bedeutung für ein Schutzkonzept, das die Risiken einer unkontrollierten Suizidbeihilfe minimieren soll, besitzt in der Konzeption des BVerfG die Feststellung der Freiverantwortlichkeit, die in ihren forensischen Aspekten allerdings noch der Konkretisierung bedarf. Dazu wird im folgenden Beitrag aus psychiatrischer Sicht Stellung genommen, um die Perspektiven unseres Faches in den Fortgang der Diskussion einzubringen.

## Abstract (en)

The verdict of the German Federal Constitutional Court on assisted suicide has led to lively debates in the medical world, in the German Federal Parliament and the civil society. In the conception of the German Federal Constitutional Court the determination of free responsibility is of central importance for a protection concept that aims to minimize an uncontrolled assistance to suicide; however, the forensic aspects of this task need to be substantiated. This article comments on this from a psychiatric aspect in order to include the perspective of our discipline in the progression of the discussion.

## Ausgangslage

Das Bundesverfassungsgericht (BVerfG) hat in seinem grundlegenden Urteil vom 26.02.2020 (BVerfGE 153, 182 ff), mit dem es den 2015 in Kraft getretenen § 217 Strafgesetzbuch (StGB) zur Strafbarkeit der geschäftsmäßigen Suizidassistenz für nichtig erklärt hat, zugleich betont, dass die Freiverantwortlichkeit dieser Entscheidung eine unabdingbare Voraussetzung für die Zulässigkeit von Suizidassistenz ist. In Übereinstimmung mit dem Europäischen Gerichtshof für Menschenrechte (EGMR) führt das BVerfG aus, der Staat habe dafür *Sorge zu tragen* und *sicherzustellen*, dass der Entschluss, assistierten Suizid zu begehen, tatsächlich auf einem freien Willen beruht (Randnummern 232 und 305 im genannten Urteil). Wie das konkret geschehen kann und soll, ist bisher (Juli 2022) nicht gesetzlich geregelt worden. Dazu haben die Autoren des vorliegenden Kapitels Diskussionsbeiträge publiziert, auf die Bezug genommen wird [6, 7]. Der Vorstand der Deutschen Gesellschaft für Psychiatrie, Psychotherapie und Nervenheilkunde (DGPPN) hat mit einer Expertenrunde verschiedene Optionen diskutiert und am 01.06.2022 ein Eckpunktepapier vorgelegt [11]. Darüber hinaus gibt es neben drei Gesetzentwürfen von Parlamentariern die Thesen und Empfehlungen der Nationalen Akademie der Wissenschaften Leopoldina vom Juli 2021 (an denen auch der vormalige Präsident des BVerfG Andreas Voßkuhle mitgewirkt hat [15]), ferner einen Diskussionsentwurf des Bundesministeriums für Gesundheit (BMG) [1]. Der vorliegende Beitrag erörtert den neuen Rechtsbegriff aus forensisch-psychiatrischer Perspektive, wobei er sich insbesondere an den vom BVerfG formulierten Grundsätzen, an den Thesen und Empfehlungen der Leopoldina sowie an dem Eckpunktepapier der DGPPN orientiert, im Übrigen die Auffassung der Autoren wiedergibt.

## Zur Definition von Freiverantwortlichkeit

Als notwendige Voraussetzungen für eine freie Suizidentscheidung hat das BVerfG vier Komponenten benannt:

- die Fähigkeit, seinen Willen frei und unbeeinflusst von einer akuten psychischen Störung zu bilden und nach dieser Einsicht zu handeln (Rn 241, 245),
- die tatsächliche Informiertheit über alle entscheidungserheblichen Gesichtspunkte (Rn 242, 246),
- die Freiheit von unzulässiger Einflussnahme oder Druck (Rn 243, 235, 247, 250),
- die Dauerhaftigkeit und innere Festigkeit des Entschlusses (Rn 244, 340).

Nachdem diese vier Komponenten integrale Bestandteile einer freien Suizidentscheidung im Sinne des BVerfG sind, eignet sich unseres Erachtens der Begriff Freiverantwortlichkeit als *Oberbegriff* für die Gesamtheit dieser Voraussetzungen. Der Begriff Freiverantwortlichkeit wurde bereits früher vom Bundesgerichtshof (BGH) im Zusammenhang mit der Lebensbeendigung gem. §§ 216, 217 a. F. StGB verwendet (z. B. BGH 04.07.1984 – 3 StR 96/84, Rn 12, 20, 22; BGH 03.07.2019 – 5 StR 132/18, Rn 20 ff), wird im Urteil des BVerfG vom 26.02.2020 in Rn 222 und 335 erwähnt und hat sich seither im Kontext der Suizidassistenz eingebürgert (z. B. [4, 6, 11, 15, 16]).

Das BVerfG hat deutlich gemacht, dass Freiverantwortlichkeit nicht nur das Freisein von einer psychischen Störung bedeutet, sondern zugleich die Dauerhaftigkeit und innere Festigkeit des Entschlusses, die hinreichende Informiertheit (Aufklärung, Beratung) sowie das Freisein von Druck bzw. Pressionen erfordert. Freiverantwortlichkeit kann auch fehlen, wenn keine psychische Störung vorliegt. Sie kann aber auch gegeben sein, wenn eine psychische Störung vorhanden ist.

Aus Gründen konzeptioneller und begrifflicher Klarheit sollte der Begriff der Freiverantwortlichkeit abgegrenzt werden gegenüber anderen Rechtsbegriffen wie *freie Willensbestimmung* (Geschäftsfähigkeit, § 104 Nr. 2 BGB), *Einwilligungsfähigkeit* (§ 630d BGB) und *Schuldfähigkeit* (§§ 20, 21 StGB), für die trotz partieller Überlappungen andere Voraussetzungen gelten. Die vom BVerfG benannten Voraussetzungen für die Freiverantwortlichkeit einer Suizidentscheidung sind nicht auf andere Sachverhalte übertragbar.

## Zur Bedeutung der Heterogenität der Betroffenen

In der öffentlichen Debatte wird meist nicht zwischen den sehr unterschiedlichen Gruppen differenziert, die einen assistierten Suizid wünschen. Anders als andere Länder hat das deutsche BVerfG die Zulässigkeit des assistierten Suizids nicht auf Personen mit unheilbaren, tödlich verlaufenden Krankheiten oder schweren Leidenszuständen beschränkt, allerdings hat es in Rn 340 je nach Lebenssituation unterschiedliche Anforderungen an den Nachweis der Ernsthaftigkeit und Dauerhaftigkeit eines Selbsttötungswillens vorgesehen. In verschiedenen Voten wird nachvollziehbar darauf hingewiesen, dass bei Suizidwünschen von Menschen mit schweren, lebenslimitierenden Erkrankungen und bei anderen Gruppen *unterschiedlich* vorgegangen werden sollte (z. B. [9, 10]; vgl. Leopoldina Empfehlung 5; BMG 2021 – Sterbehilfegesetz (StHG) § 7; [6, Rn 11]). Nach psychiatrischer Erfahrung und den national und international vorliegenden Daten lassen sich folgende vier Hauptgruppen von Suizidwilligen unterscheiden:

### 1. Spontansuizide

Die Gruppe der Menschen, die sich unabhängig von irgendwelchen gesetzlichen Regelungen bisher ohne Hilfe Dritter auf teilweise brutale Weise das Leben genommen haben, spielt in der gegenwärtigen Debatte eine besondere Rolle, weil die Erwartung besteht, dass die Freigabe der Suizidhilfe diesen Menschen zu einem sanfteren Tod verhilft und zugleich Gefährdungen Dritter reduziert (z. B. Auto‑, Flugzeug- und Bahnsuizide etc.). Wie die Entwicklung in den Ländern zeigt, die die Suizidassistenz oder gar die Tötung auf Verlangen liberalisiert haben, hat sich die genannte Erwartung dort nicht erfüllt: Die Liberalisierung der Suizidassistenz geht nicht mit einem signifikanten Rückgang der Spontansuizide einher, sondern teilweise sogar mit deren Zunahme ([3, S. 24 ff.]; vgl. [19, S. 121, 134 Ziff. 7]). Tragischerweise fehlt vielen der Betroffenen gerade in den schwersten Krankheitszuständen die Einsicht, dass ihr Zustand krankheitsbedingt ist und behandelbar wäre. Es ist davon auszugehen, dass die meisten von ihnen nicht um Suizidassistenz nachsuchen und daher auch nicht zur Beurteilung ihrer Freiverantwortlichkeit kommen werden. Entscheidend für diese Personengruppe ist die Verbesserung der Suizidprävention (vgl. [8, 14]).

### 2. Unheilbar terminal Kranke

Diese in der öffentlichen Debatte stark beachtete Gruppe wird meist als paradigmatisch für die Humanität der Suizidassistenz angeführt. Es besteht Konsens, dass terminal Kranken eine bestmögliche Linderung ihrer Schmerzen und Leiden gewährt werden soll, selbst wenn dies zu einer Lebensverkürzung führt. Auch vor dem Urteil des BVerfG war es unstreitig, dass die Betroffenen lebenserhaltende Maßnahmen ablehnen und Behandlungen abbrechen können. Große Fortschritte der Schmerztherapie, der Palliativmedizin sowie der Hospizangebote haben zu einer wesentlichen Verbesserung der Situation körperlich Schwerkranker an ihrem Lebensende geführt. Wie hoch der Prozentsatz der Personen ist, die durch diese Angebote nicht hinreichend Erleichterung finden oder diese ablehnen und den Eintritt ihres Todes durch einen assistierten Suizid beschleunigen wollen, ist nicht bekannt. Dasselbe gilt für die Lebensumstände und die Gründe, die sie zu diesem Wunsch veranlassen. Die auch vom BVerfG geäußerte Befürchtung (Rn 243, 247, 250), dass ein allgemeiner gesellschaftlicher Druck oder aber auch konkrete Pressionen auf diese Personen ausgehen können, das Leben vorzeitig zu beenden, um den Angehörigen oder der Gesellschaft Belastungen bzw. Kosten zu ersparen, gilt sicherlich in besonderem Maße für diese Personengruppe.

Über die psychische Situation und evtl. psychische Störungen bei terminal kranken Menschen mit Suizidwunsch ist wissenschaftlich wenig bekannt, aber die Annahme liegt nahe, dass viele von ihnen an depressiven und/oder Angst- bzw. Belastungsstörungen und manche an altersassoziierten kognitiven Beeinträchtigungen leiden, je nach körperlicher Grunderkrankung oder Medikation möglicherweise auch an Störungen der Vigilanz bzw. des Bewusstseins.

Wenn durch Fachärzte des entsprechenden medizinischen Gebiets und/oder durch Palliativmediziner eine terminale Krankheit mit einer zu erwartenden Lebensdauer von allenfalls wenigen Monaten nachgewiesen ist, sollten gemäß Rn 340 des BVerfG-Urteils geringere Anforderungen an den Nachweis der Ernsthaftigkeit und Dauerhaftigkeit des Selbsttötungswillens gestellt werden. Im Falle depressiver oder ängstlicher Syndrome bzw. anderer psychischer Auffälligkeiten sollte ein Facharzt für Psychiatrie und Psychotherapie zur Prüfung etwa noch vorhandener Therapieoptionen hinzugezogen werden. Ansonsten kann die Prüfung der Freiverantwortlichkeit in diesen Fällen auch durch die behandelnden Ärzte erfolgen, um die Belastung für die tödlich Kranken möglichst gering zu halten.

### 3.Kranke mit therapieresistenten unerträglichen Leidenszuständen

Dieser Begriff taucht in den entsprechenden Regelungen und Gesetzestexten anderer Länder auf. Hier geht es beispielsweise um tief in die Lebensführung eingreifende somatische Erkrankungen, um schwere, chronische Schmerzsyndrome, um ein breites Spektrum hartnäckiger somatoformer und hypochondrischer Störungsbilder und schließlich auch, was besonders vorsichtig zu bewerten wäre, um gravierende psychische Erkrankungen mit andauernd massiver Beeinträchtigung von Lebensqualität und sozialen Bezügen. Suizidwillige aus dieser Gruppe sollten zunächst von Fachärzten des zuständigen Spezialgebiets daraufhin untersucht werden, ob alle nach dem Stand der Wissenschaft gebotenen Therapieoptionen und Unterstützungsmöglichkeiten genutzt wurden. Außerdem sollte durch diesbezüglich besonders erfahrene Fachärzte für Psychiatrie und Psychotherapie oder Schmerztherapeuten geklärt werden, welche psychotherapeutischen und/oder medikamentösen Behandlungsangebote den Betroffenen vielleicht doch noch Linderung bringen könnten. Wenn diese Behandlungsoptionen erschöpft sind bzw. keine Linderung versprechen, sollte im Rahmen einer abgekürzten Wartefrist die Freiverantwortlichkeit fachärztlich überprüft werden.

### 4. Suizidwillige, bei denen die Voraussetzungen der Gruppen 2 und 3 nicht vorliegen

Zu dieser großen Gruppe gehören zum einen Menschen, die unter einer psychischen Störung leiden, zum anderen all jene, die aufgrund von Schicksalsschlägen, anderen belastenden Lebensereignissen, Lebens- oder Sinnkrisen, Lebensüberdruss oder aus sonstigen Gründen einen assistierten Suizid wünschen. Für diese Personengruppen sollte nach eingehender Aufklärung und Beratung über medizinische oder psychosoziale Hilfen und Alternativen zum Suizid sowie einer Vorprüfung der Freiverantwortlichkeit eine Wartefrist von i. d. R.* sechs Monaten* eingehalten werden, nach deren Ablauf die Freiverantwortlichkeit durch zwei Ärzte zu überprüfen ist, von denen mindestens einer Facharzt für Psychiatrie und Psychotherapie sein muss.

## Beurteilung der Freiverantwortlichkeit

Unabhängig von der begrifflichen Frage, ob Freiverantwortlichkeit als Oberbegriff oder als eine der vier genannten Komponenten verstanden werden soll, lässt sich darlegen, welche *Kriterien *das Urteil des BVerfG für eine gemäß Rn 241–244 freie Suizidentscheidung insgesamt nahelegt. Diese sollen im Folgenden aus psychiatrischer Sicht erörtert werden, wobei allerdings zu betonen ist, dass es beim gegenwärtigen Stand der Diskussion und beim Ausstehen gesetzlicher oder anderer Regelungen zur Ausfüllung des Schutzkonzeptes noch nicht um eine detaillierte Begutachtungsanleitung gehen kann.

### Zum Kriterium, den Willen frei und unbeeinflusst durch eine psychische Störung zu bilden

Entsprechend der *ersten Beurteilungsebene* in der üblicherweise zweistufigen Begutachtungsmethodik (vgl. [5, S. 37]) geht es hier um die Frage, ob bei der betroffenen Person eine psychische Störung vorliegt. Dies entspricht dem Vorgehen, das bei allen psychiatrischen Begutachtungen in Rechtsfragen üblich ist, wobei jeweils der für das betreffende Gutachtensthema *rechtlich* vorgegebene Störungsbegriff zugrunde zu legen ist (vgl. [18]).

Hier sind zunächst alle psychischen Störungen in Betracht zu ziehen, die auch bei der Beurteilung der freien Willensbestimmung im Sinne der §§ 104, 105, 2229 BGB, der Einwilligungsfähigkeit gemäß § 630d BGB oder der Schuldfähigkeit gemäß §§ 20, 21 StGB zu berücksichtigen sind (vgl. [5, 13, 17]). Von besonderer Bedeutung ist allerdings, dass im vorliegenden Kontext – anders als etwa bei der Geschäfts- oder Testierfähigkeit – ein weiterer Störungsbegriff zugrunde zu legen ist. Nachvollziehbar begründet nämlich das BVerfG die besonders hohen Anforderungen an die *Freiverantwortlichkeit* im Hinblick auf einen assistierten Suizid damit, dass wegen der Unumkehrbarkeit des Vollzugs der Suizidentscheidung und des hohen Rechtsguts Leben die Betroffenen sowie ihr soziales Umfeld soweit wie irgend möglich vor irreversiblen Fehlentscheidungen bewahrt werden müssen, weshalb auch *vorübergehende Lebenskrisen* und *Fehlvorstellungen* auszuschließen sind (Rn 244, 246).

Das bedeutet, dass hier kein enger Krankheits-, sondern ein weitgefasster Störungsbegriff anzuwenden ist, der auch vorübergehende Lebenskrisen (Belastungsreaktionen, Anpassungsstörungen u. Ä.) mit umfasst. Deshalb kommen auf der ersten, diagnostischen Beurteilungsebene praktisch alle Störungen der Kategorie F der ICD-10 in Betracht, die geeignet sind, die Entscheidungsfreiheit bezüglich eines assistierten Suizids einzuschränken, insbesondere auch solche, die erfahrungsgemäß vorübergehender Natur bzw. mit Aussicht auf Erfolg behandelbar sind. Diese müssen unter dem Aspekt der geforderten Dauerhaftigkeit des Suizidwunsches zunächst angemessen behandelt oder es muss die Möglichkeit einer Spontanremission ausreichend lange abgewartet werden. Darüber hinaus sind bei schweren körperlichen Erkrankungen auch Apathiesyndrome und ihre Auswirkungen auf die Motivation zu berücksichtigen [12].

Auf der *ersten, diagnostischen Beurteilungsebene* führt das Vorliegen einer psychischen Störung allein nicht zwangsläufig zum Fehlen von Freiverantwortlichkeit, vielmehr ist ggf. auf der *zweiten Beurteilungsebene* zu prüfen, ob und auf welche Weise die psychopathologischen Phänomene der vorliegenden Störung die Entscheidungsfreiheit für einen assistierten Suizid beeinträchtigen. Zu berücksichtigen ist dabei allerdings, wie ausgeführt, dass es im Kontext der Freiverantwortlichkeit nicht um den engen Krankheitsbegriff im zivilrechtlichen Sinne geht, sondern dass hier von dem weiteren Störungsbegriff auszugehen ist, den das BVerfG in seinem Urteil vom 26.02.2020 umrissen hat. So können beispielsweise depressive Syndrome, Angstsyndrome, Apathie, Abhängigkeitserkrankungen und Schmerzsyndrome das Bewusstsein und die Abwägungsfähigkeit für alternative Optionen zum Suizid erheblich einengen (*affektive Vereinseitigung*, vgl. [5, S. 88]). Die psychiatrisch-psychotherapeutische Erfahrung zeigt, dass der Verlust wichtiger Bezugspersonen durch Trennung oder Tod sowie gravierende Enttäuschungen, Misserfolge oder Kränkungen häufig mit dem Gefühl einhergehen, unter diesen Umständen nicht mehr weiterleben zu können oder zu wollen, wobei sich die betroffenen Menschen zunächst nicht vorstellen können oder wollen, dass dieser Zustand gefühlter Alternativlosigkeit in der Regel vorübergehender Natur ist und „die Welt“ danach wieder „anders aussieht“ (vgl. z. B. [2, S. 219, 20]). Nachvollziehbar geht das BVerfG davon aus, dass auch solche, i. d. R. vorübergehende Lebenskrisen oder Fehlvorstellungen die freiverantwortliche Entscheidung für den irreversiblen Akt der Selbsttötung beeinträchtigen können (Rn 244, 246).

### Zum Kriterium der Informiertheit, Aufklärung, Beratung

Hierzu heißt es im Urteil:

„Erforderlich ist, dass [der Suizidwillige] über sämtliche Informationen verfügt, er also in der Lage ist, auf einer hinreichenden Beurteilungsgrundlage realitätsgerecht das Für und Wider abzuwägen. Eine freie Willensbildung setzt hierbei insbesondere voraus, dass der Entscheidungsträger Handlungsalternativen zum Suizid erkennt, ihre jeweiligen Folgen bewertet und seine Entscheidung in Kenntnis aller erheblichen Umstände und Optionen trifft. Insoweit gelten dieselben Grundsätze wie bei einer Einwilligung in eine Heilbehandlung“ (BVerfGE 153, 182 ff, Rn 242).

Bei diesem an der *Einwilligungsfähigkeit* orientierten Merkmalskomplex geht es also um die Sicherstellung des für eine freiverantwortliche Entscheidung notwendigen Wissens über die ggf. vorliegende körperliche Erkrankung, die psychische Störung oder seelische Krise und/oder die sonstigen Problemlagen sowie über die hierfür real existierenden Hilfemöglichkeiten medizinischer oder anderer Art (z. B. sozialpädagogische, Rechts‑, Mieter- oder Schuldnerberatung) als Alternativen zu einem assistierten Suizid. Anzustreben ist, den in diesen Fällen häufig eingeengten Blickwinkel zu erweitern und neue Perspektiven zu eröffnen, auch *Fehlvorstellungen* entgegenzuwirken (Rn 246), etwa irrigen Annahmen über die Prognose und Behandlungsmöglichkeiten der vorliegenden Erkrankung.

Zunächst sollte daher vom jeweiligen Ansprechpartner vorsichtig geprüft werden, von welcher Informationslage der Betroffene ausgeht, ob diese realistisch und ausreichend ist, wo evtl. Ergänzungen oder Korrekturen erforderlich sind, insbesondere auch zu den nach aktuellem Kenntnisstand optimalen Behandlungsmöglichkeiten einschließlich palliativer und Hospizangebote. Beim psychiatrisch-psychotherapeutischen Erstgespräch sollte das Feld umsichtig sondiert und es sollten die für die individuelle Problemlage bestgeeigneten Fachleute vermittelt werden, die dann die weitere fachspezifische Beratung und Aufklärung übernehmen (vgl. Empfehlungen 2 bis 4 der Leopoldina [15]).

### Zum Kriterium des Freiseins von unzulässigen Einflussnahmen oder Druck

Im Rahmen der Überprüfung der Freiverantwortlichkeit und bei der Aufklärung und Beratung sollte in *allen* Fallgruppen stets auch geprüft werden, ob es Hinweise gibt, dass die Suizidentscheidung unter subjektiv empfundenem bzw. tatsächlich ausgeübtem sozialem Druck zustande gekommen ist. In diesen Fällen sollten individuelle Möglichkeiten der Entlastung gesucht und auch die Bezugspersonen einbezogen werden. Zutreffend spricht die Leopoldina in ihrer 4. Empfehlung diesbezüglich von *gefühltem oder realem äußeren Druck. *Damit wird die *subjektive* Dimension dieses Merkmals deutlich, auf die bei den Beratungsgesprächen besondere Aufmerksamkeit zu richten ist. Dies gilt insbesondere auch für die psychiatrisch-psychotherapeutischen Explorationen, wobei darauf hinzuweisen ist, dass in der therapeutischen wie gutachterlichen Tätigkeit in unserem Fach eine umfangreiche Expertise zu diesem Fragenkomplex erworben wird.

### Zum Kriterium der Dauerhaftigkeit/inneren Festigkeit des Entschlusses

Vom BVerfG wird als weiteres wesentliches Beurteilungskriterium die *Dauerhaftigkeit/innere Festigkeit* der Entscheidung gefordert, wobei ausdrücklich auf vorübergehende Lebenskrisen verwiesen wird (Rn 244). In Übereinstimmung mit dem Diskussionsentwurf des BMG [1] sollte u. E. die Beobachtungs- bzw. Bedenkzeit jedenfalls in Fällen der oben beschriebenen Gruppe 4* mindestens sechs Monate* betragen. Bei den oben aufgeführten Gruppen 2 und 3 können und sollten je nach den individuellen Gegebenheiten kürzere Fristen festgelegt werden (BVerfG Rn 340).

Der im Gesetzentwurf von Castellucci et al. (Stand März 2022) vorgesehene Mindestabstand von 3,5 Monaten ist aus unserer Sicht unzureichend, weil die psychiatrisch-psychotherapeutische Erfahrung zeigt, dass die zu Suizidabsichten Anlass gebenden schweren Lebenskrisen (z. B. Verlust einer nahen Bezugsperson, Scheidung, Querschnittslähmung, berufliches Scheitern) für ihre Bewältigung und die Neuorientierung häufig sechs Monate oder mehr benötigen. Die Deutsche Gesellschaft für Palliativmedizin [9] und die Deutsche Gesellschaft für Pneumologie und Beatmungsmedizin [10] empfehlen im Allgemeinen sogar mindestens ein Jahr Abstand.

## Zu den Beweisanforderungen

Das BVerfG hat in Übereinstimmung mit dem EGMR betont, dass der Staat *sicherzustellen* hat, dass der Entschluss für einen assistierten Suizid *tatsächlich auf einem freien Willen beruht* (Rn 232, 305). Ähnlich betont die Leopoldina, dass *sichergestellt *sein muss, dass psychische oder andere, insbesondere medizinische Gründe *nicht vorliegen*, die eine autonome Suizidentscheidung ernsthaft infrage stellen; dies bedürfe zwingend ärztlicher Expertise.

Im Gegensatz zur Begutachtung der Geschäfts- und Testierfähigkeit, wo das *Fehlen* der freien Willensbstimmung nachgewiesen werden muss, muss im Kontext der Suizidassistenz *positiv* festgestellt werden, dass die Suizidentscheidung mit hinreichender Sicherheit freiverantwortlich getroffen wurde bzw. dass keine Gründe für ernsthafte Zweifel an der Freiverantwortlichkeit vorliegen. Dabei muss nach unserer Auffassung wie bei anderen Begutachtungen auch die Gesamtwürdigung der in den Beratungsgesprächen und psychiatrischen Gutachten festgestellten Umstände und die abschließende Entscheidung über das Vorliegen von Freiverantwortlichkeit letztlich durch das Gericht bzw. eine dafür einzurichtende Institution getroffen werden, nicht von den Sachverständigen.

Aus forensisch-psychiatrischer Sicht nicht nachvollziehbar und mit dem Wortlaut des BVerfG-Urteils in Rn 232 und 305 u. E. auch nicht vereinbar erscheint der in den Entwürfen des BMG [1] sowie der Gruppe um Castellucci [4] jeweils vorgeschlagene Absatz 3 des § 217 StGB, wonach die Prüfung der Freiverantwortlichkeit und eine Aufklärung/Beratung der Suizidwilligen *nicht* vorgesehen ist, wenn die Suizidassistenz durch *Angehörige *oder dem Suizidwilligen *nahestehende Personen* ausgeübt wird. Während Ausnahmeregelungen im Sinne von Rn 340 des BVerfG-Urteils für bestimmte Gruppen von Suizidwilligen, insbesondere für terminal kranke Personen, vertretbar und angemessen erscheinen, erschließt sich nicht, warum bestimmte Eigenschaften der *Suizidhelfer* eine Prüfung der Freiverantwortlichkeit der Suizidwilligen entbehrlich machen sollten, zumal die Suizidhelfer ja durchaus spezifische Eigeninteressen haben können. Die vom BVerfG wiederholt betonte Gefahr von Einflussnahmen und Pressionen auf den betroffenen Personenkreis (Rn 235, 243, 247) geht erfahrungsgemäß oft gerade von überforderten Angehörigen oder interessierten Dritten aus; auch das BVerfG benennt in diesem Zusammenhang explizit das *familiäre Umfeld* (Rn 250). Aus diesem Grund dürfen nach den Vorschlägen der Leopoldina sowie der beiden hier zitierten Gesetzentwürfe die Suizidhelfer ja auch keinesfalls mit den Personen identisch sein, die den Suizidwilligen aufklären, beraten und die Voraussetzungen für Freiverantwortlichkeit prüfen. Insofern sind beide Gesetzentwürfe mit dem Absatz 3 in sich widersprüchlich.

Abschließend sei als besonders dringliches Problem benannt, dass eine *Altersbeschränkung* für die Inanspruchnahme von Suizidassistenz vom BVerfG nicht vorgesehen ist (Rn 210). Sowohl die Empfehlungen der Leopoldina als auch die Entwürfe des BMG und der Gruppe um Castellucci gehen davon aus, dass grundsätzlich nur die Entscheidung von Volljährigen als Ausdruck eines autonom gebildeten Suizidwillens anerkannt werden und nur in besonderen Fällen Ausnahmen möglich sein sollen. Dem schließen wir uns ausdrücklich an.

## Fazit

Aus psychiatrischer Sicht ist bei der grundsätzlichen Ermöglichung des assistierten Suizids die essenzielle Bedeutung eines legislativen Schutzkonzeptes zu betonen. Im besonderen Maße betrifft dies wegen ihrer erhöhten Vulnerabilität und der erhöhten Suiziddisposition die Menschen mit psychischen Erkrankungen oder Störungen. Aber darüber hinaus müssen wegen des komplexen psychosozialen Bedingungsgefüges von Suizidalität die Voraussetzungen für die Inanspruchnahme von Suizidassistenz sorgfältig eingegrenzt werden. Im Spannungsfeld zwischen Selbstbestimmung, staatlichen Schutzaufgaben und ärztlichen Fürsorgepflichten bietet das mehrdimensionale Konstrukt der Freiverantwortlichkeit u. E. einen gut geeigneten Rahmen. Die weitere Konkretisierung der vier Beurteilungsdimensionen erfordert jedoch einen intensiven Dialog der beteiligten Disziplinen und Akteure.
